# Supplementary material for: Estimating the gains of early detection of hypertension over the marginal patient
Source: PLoS One. 2021 Jul 9;16(7):e0254260. doi: 10.1371/journal.pone.0254260 (PMC8270433; doi:10.1371/journal.pone.0254260)
Supplement: S1 Appendix — (PDF) [file pone.0254260.s001.pdf]

A Sample selection

The analysis was conducted on a subset of all available data from the HSE-ELSA data. Individuals not diagnosed with HBP or diabetes and not taking BP-lowering medication were selected. In the ELSA survey design, everyone who is asked about BP medication reports being diagnosed with HBP by design of the survey. That is not the case with the HSE, where the analysis of medication is much more detailed. Even though the restriction on prior diagnosis greatly reduced the sample size, such restriction avoided the endogeneity of being below the cutoffs for individuals who were concerned about their BP. The right-hand graph in S1 Fig shows that without the filters, individuals just below the threshold were more likely to be taking BP medication (7 percentage points more likely). This result is to be expected since those respondents are aware of their BP condition; since they know their BP levels are above the cutoff increase for CVD risk, they do their best to control their BP.

Another problem is that the cutoff for HBP is the same as that for the advice. Accordingly, it is more likely that individuals above such levels will be diagnosed with the condition. The estimated jump at $t = 0$ was not significant (left-hand graph in S1 Fig), but this might have induced the jump observed at $t = 1$. Thus, at the expense of larger standard errors, the sample is restricted to only the new cases.

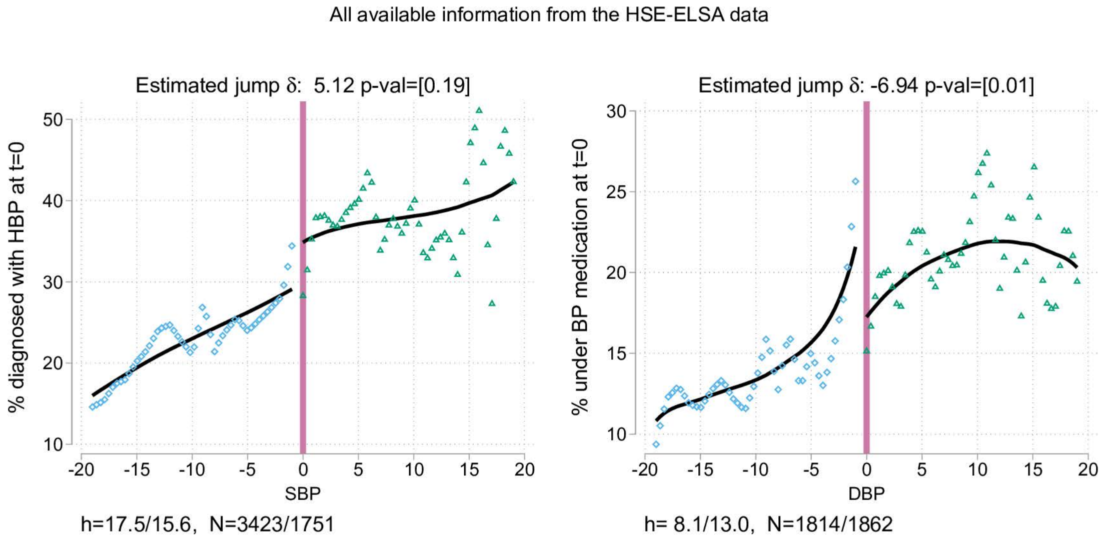

S1 Fig. HBP diagnosis and medication use according to SBP measured in the same wave

The other inclusion criteria for this study was age. S2 Fig shows that the main outcomes decreased with age. This indicates that the advice produces differences in the detection rates of HBP for younger individuals. This result is to be expected since the demand for primary care services increases with age; therefore, any difference due to the advice will last less than 2 years. The advantage of an early-detection program is that asymptomatic conditions are detected earlier; for that reason, the sample for this study was selected to be relatively young.

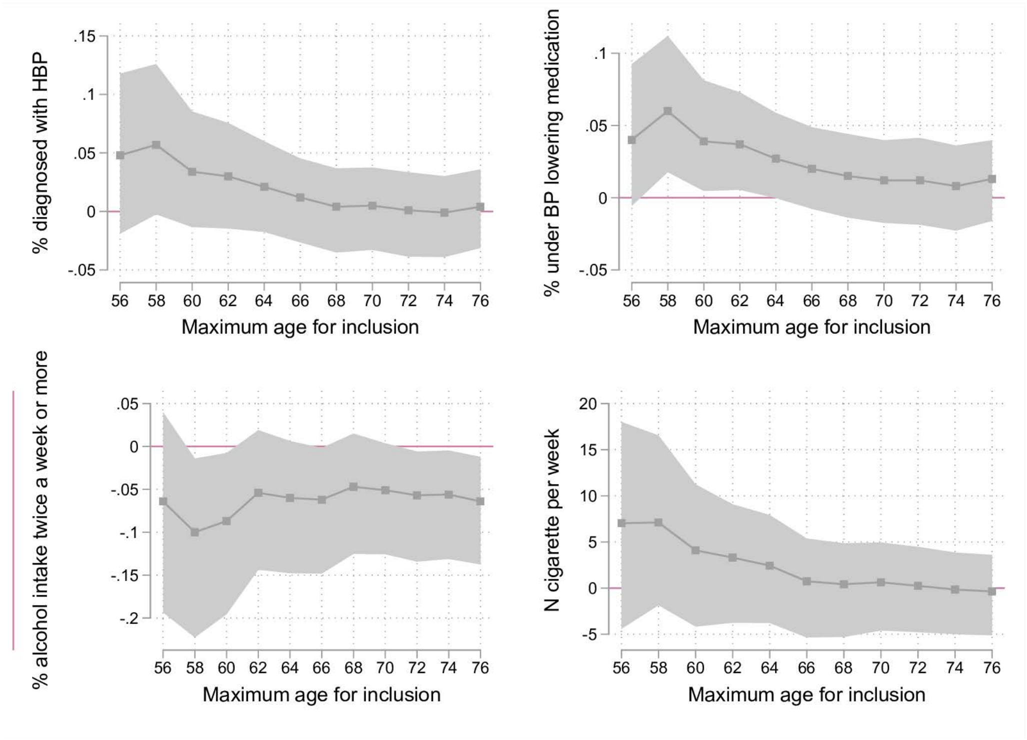

S2 Fig. Impact of nurse advice by maximum age

B Alternative specifications

Several tests were performed to confirm the quality of the results. The first was to add controls to the estimates. Table 1 presents the findings by applying the same methodology but adding as covariates (measured at $t = 0$) basic demographic controls (panel A) as well as information on health and lifestyle (panels B and C). The results are similar to the main ones, where no control was considered.

A typical concern with non-parametric estimators is their potential dependence on ad hoc parameters. In this case, the jump estimator may be very sensitive to the bandwidth selection and order of the local polynomial. Table 2 presents estimates under alternative bandwidths and Table 3 under alternative polynomial order.

Table 1. The effects of information on potential hypertension status: including covariates

Dependent Variable	Panel A: age, male, non-white, married, and education level dummies<br>Before BP test<br>$t=0$	 2 years<br>$t=1$	 4 years<br>$t=2$	Panel B: + being on good health, BMI<br>Before BP test<br>$t=0$	 2 years<br>$t=1$	 4 years<br>$t=2$	Panel C: + current smoker, alcohol intake twice a week or more<br>Before BP test<br>$t=0$	 2 years<br>$t=1$	 4 years<br>$t=2$
**Panel A. Attrition**									
Missing this wave		0.006<br>(0.030)[0.815]<br>h=10.4/11.8<br>N=1328/867	0.048<br>(0.038)[0.191]<br>h= 7.9/14.2,<br>N=906/1013		−0.008<br>(0.028)[0.771]<br>h=12.7/12.5<br>N=1617/883	0.023<br>(0.034)[0.428]<br>h=10.2/14.6,<br>N=1257/966		−0.009<br>(0.030)[0.822]<br>h=13.1/11.7<br>N=1091/505	−0.016<br>(0.040)[0.754]<br>h=12.1/11.6,<br>N=1004/505
Already death by this wave		−0.003<br>(0.005)[0.415]<br>h= 7.2/ 6.1<br>N=832/526	0.000<br>(0.008)[0.734]<br>h=14.8/10.9,<br>N=1813/745		−0.003<br>(0.005)[0.470]<br>h= 7.1/ 5.5<br>N=791/457	0.000<br>(0.009)[0.780]<br>h=14.4/10.8,<br>N=1733/715			0.008<br>(0.010)[0.455]<br>h=15.7/20.3,<br>N=1116/609
**Panel B. High Blood Pressure**									
Diagnosed HBP ever		0.056<br>(0.026)[0.073]<br>h=15.7/14.2<br>N=1827/834	0.038<br>(0.035)[0.410]<br>h=17.7/14.8,<br>N=1846/750		0.052<br>(0.025)[0.074]<br>h=17.6/16.0<br>N=2026/849	0.034<br>(0.035)[0.498]<br>h=19.0/15.3,<br>N=1928/745		0.071<br>(0.032)[0.033]<br>h=11.1/10.8<br>N=823/419	0.053<br>(0.041)[0.178]<br>h=13.7/13.0,<br>N=899/454
Takes BP medication		0.059<br>(0.019)[0.009]<br>h=14.2/12.0<br>N=1687/758	0.036<br>(0.026)[0.237]<br>h=17.0/12.3,<br>N=1849/671		0.058<br>(0.020)[0.014]<br>h=17.8/11.5<br>N=2026/688	0.039<br>(0.027)[0.207]<br>h=16.4/12.4,<br>N=1663/644		0.053<br>(0.021)[0.026]<br>h= 6.9/12.1<br>N=383/476	0.048<br>(0.028)[0.078]<br>h=13.1/12.9,<br>N=908/437
**Panel C. Health**									
Diagnosed Diabetes ever		0.012<br>(0.011)[0.284]<br>h=14.7/13.1<br>N=1687/791	0.018<br>(0.019)[0.339]<br>h=18.5/13.9,<br>N=1976/694		0.003<br>(0.010)[0.788]<br>h=13.9/12.5<br>N=1494/726	0.010<br>(0.019)[0.560]<br>h=15.4/13.8,<br>N=1541/666		−0.005<br>(0.012)[0.626]<br>h=18.3/12.8<br>N=1468/476	0.020<br>(0.023)[0.482]<br>h=19.0/12.6,<br>N=1312/433
Diagnosed Heart Condition	0.001<br>(0.009)[0.989]<br>h=14.2/13.4,<br>N=1986/961	0.022<br>(0.015)[0.255]<br>h=14.3/19.8<br>N=1688/970	0.038<br>(0.021)[0.119]<br>h=15.4/13.3,<br>N=1601/701	−0.005<br>(0.009)[0.538]<br>h=15.2/11.3,<br>N=2058/829	0.016<br>(0.016)[0.459]<br>h=14.3/16.8<br>N=1620/850	0.026<br>(0.020)[0.249]<br>h=15.7/14.4,<br>N=1548/707	0.001<br>(0.012)[0.892]<br>h=15.9/12.9,<br>N=1281/533	−0.005<br>(0.020)[0.636]<br>h=11.9/14.3<br>N=823/522	−0.009<br>(0.020)[0.567]<br>h=17.2/15.4,<br>N=1219/497
Self-reported GOOD health	0.008<br>(0.033)[0.737]<br>h=11.8/18.2,<br>N=1513/1142	−0.020<br>(0.034)[0.609]<br>h=13.2/16.9<br>N=1550/886	−0.003<br>(0.043)[0.949]<br>h=10.5/13.5,<br>N=979/696		−0.021<br>(0.028)[0.522]<br>h=15.5/17.0<br>N=1749/874	0.003<br>(0.037)[0.854]<br>h=11.2/15.3,<br>N=1074/727		−0.026<br>(0.034)[0.318]<br>h=13.8/10.9<br>N=1000/418	−0.026<br>(0.038)[0.575]<br>h=16.2/14.6,<br>N=1133/478
Systolic BP	−0.268<br>(0.445)[0.524]<br>h=15.0/13.1,<br>N=2148/964		0.401<br>(1.569)[0.972]<br>h=18.6/15.4,<br>N=1485/580	−0.024<br>(0.427)[0.958]<br>h=17.9/13.9,<br>N=2374/921		0.055<br>(1.594)[0.846]<br>h=18.8/15.0,<br>N=1440/536	0.286<br>(0.488)[0.594]<br>h=13.3/15.9,<br>N=1091/606		−0.124<br>(1.718)[0.789]<br>h=16.1/11.7,<br>N=873/314
Diastolic BP	−0.951<br>(0.652)[0.278]<br>h=13.6/14.6,<br>N=1844/1014		−0.280<br>(1.080)[0.869]<br>h=12.8/14.2,<br>N=949/561	−1.028<br>(0.649)[0.215]<br>h=14.7/14.1,<br>N=1902/966		−0.293<br>(1.075)[0.859]<br>h=13.1/13.2,<br>N=986/509	−1.285<br>(0.771)[0.189]<br>h=16.3/15.8,<br>N=1375/606		−0.993<br>(1.122)[0.373]<br>h=17.0/13.6,<br>N=873/345
Body Mass Index	0.140<br>(0.410)[0.598]<br>h=14.8/12.7,<br>N=1902/883		0.822<br>(0.561)[0.142]<br>h=13.8/ 9.8,<br>N=1145/471			0.068<br>(0.195)[0.865]<br>h=17.5/13.2,<br>N=1497/577			0.012<br>(0.228)[0.953]<br>h=13.3/16.0,<br>N=778/435

Notes: This table present the impact estimates under several three specifications of a regression discontinuity design over the systolic blood pressure of respondents centred around 140 mmHg for ELSA. For males aged 50 and older in the HSE, the standardisation is done around 160 mmHg due to a different measurement protocol. It includes only those respondents aged 58 or younger at the time of the measurement who were not diagnosed with HBP or diabetes, and not taking BP-lowering medication. h presents the optimal bandwidth to the left/right of the cutoff, and N the corresponding number of observations effectively included.
Standard errors, in parenthesis, are derived from heteroskedasticity-robust nearest neighbour variance estimator with at least 3 neighbours. In the first set of brackets the conventional p-value is presented, while the second corresponds to the robust inference version derived by Calonico, Cattaneo, Titiunik (2014).

Table 2. The effects of information on potential hypertension status: fixed bandwidth

Dependent Variable	h=10<br>Before BP test<br>$t=0$	 2 years<br>$t=1$	 4 years<br>$t=2$	h=14<br>Before BP test<br>$t=0$	 2 years<br>$t=1$	 4 years<br>$t=2$	h=18<br>Before BP test<br>$t=0$	 2 years<br>$t=1$	 4 years<br>$t=2$
**Panel A. Attrition**									
Missing this wave		0.014<br>(0.034)[0.053]<br>h=10.0/10.0,<br>N=1194/768	0.041<br>(0.043)[0.087]<br>h=10.0/10.0<br>N=1192/767		−0.003<br>(0.029)[0.347]<br>h=14.0/14.0,<br>N=1894/980	0.016<br>(0.036)[0.196]<br>h=14.0/14.0<br>N=1892/979		−0.004<br>(0.026)[0.717]<br>h=18.0/18.0,<br>N=2530/1131	0.006<br>(0.032)[0.342]<br>h=18.0/18.0<br>N=2527/1130
Already death by this wave		−0.004<br>(0.004)[0.704]<br>h=10.0/10.0,<br>N=1097/704	−0.009<br>(0.011)[0.284]<br>h=10.0/10.0<br>N=1097/704		−0.002<br>(0.004)[0.313]<br>h=14.0/14.0,<br>N=1730/903	−0.004<br>(0.008)[0.296]<br>h=14.0/14.0<br>N=1730/903		−0.002<br>(0.003)[0.651]<br>h=18.0/18.0,<br>N=2323/1043	−0.003<br>(0.007)[0.561]<br>h=18.0/18.0<br>N=2323/1043
**Panel B. High Blood Pressure**									
Diagnosed HBP ever		0.060<br>(0.033)[0.498]<br>h=10.0/10.0,<br>N=1015/637	0.035<br>(0.047)[0.876]<br>h=10.0/10.0<br>N=888/569		0.060<br>(0.028)[0.158]<br>h=14.0/14.0,<br>N=1603/803	0.038<br>(0.039)[0.621]<br>h=14.0/14.0<br>N=1401/722		0.056<br>(0.024)[0.077]<br>h=18.0/18.0,<br>N=2155/931	0.038<br>(0.034)[0.510]<br>h=18.0/18.0<br>N=1886/837
Takes BP medication		0.061<br>(0.022)[0.218]<br>h=10.0/10.0,<br>N=1015/637	0.033<br>(0.031)[0.862]<br>h=10.0/10.0<br>N=889/560		0.058<br>(0.018)[0.025]<br>h=14.0/14.0,<br>N=1603/803	0.035<br>(0.026)[0.444]<br>h=14.0/14.0<br>N=1402/707		0.049<br>(0.016)[0.004]<br>h=18.0/18.0,<br>N=2155/931	0.027<br>(0.023)[0.249]<br>h=18.0/18.0<br>N=1888/819
**Panel C. Health**									
Diagnosed Diabetes ever		0.014<br>(0.013)[0.470]<br>h=10.0/10.0,<br>N=1015/637	0.017<br>(0.025)[0.830]<br>h=10.0/10.0<br>N=883/555		0.011<br>(0.011)[0.322]<br>h=14.0/14.0,<br>N=1603/803	0.017<br>(0.020)[0.610]<br>h=14.0/14.0<br>N=1389/702		0.008<br>(0.010)[0.303]<br>h=18.0/18.0,<br>N=2155/931	0.013<br>(0.018)[0.414]<br>h=18.0/18.0<br>N=1873/814
Diagnosed Heart Condition	0.001<br>(0.012)[0.727]<br>h=10.0/10.0,<br>N=1193/765	0.015<br>(0.021)[0.668]<br>h=10.0/10.0,<br>N=1015/638	0.033<br>(0.026)[0.246]<br>h=10.0/10.0<br>N=887/562	−0.002<br>(0.009)[0.764]<br>h=14.0/14.0,<br>N=1892/977	0.018<br>(0.017)[0.620]<br>h=14.0/14.0,<br>N=1603/804	0.034<br>(0.022)[0.259]<br>h=14.0/14.0<br>N=1395/710	0.001<br>(0.008)[0.881]<br>h=18.0/18.0,<br>N=2528/1127	0.022<br>(0.015)[0.575]<br>h=18.0/18.0,<br>N=2156/932	0.034<br>(0.019)[0.220]<br>h=18.0/18.0<br>N=1882/823
Self-reported GOOD health	0.010<br>(0.039)[0.745]<br>h=10.0/10.0,<br>N=1194/768	−0.015<br>(0.042)[0.608]<br>h=10.0/10.0,<br>N=1005/636	−0.004<br>(0.048)[0.901]<br>h=10.0/10.0<br>N=882/557	0.004<br>(0.032)[0.730]<br>h=14.0/14.0,<br>N=1894/980	−0.013<br>(0.035)[0.653]<br>h=14.0/14.0,<br>N=1591/801	−0.008<br>(0.039)[0.936]<br>h=14.0/14.0<br>N=1390/704	−0.001<br>(0.029)[0.731]<br>h=18.0/18.0,<br>N=2530/1131	−0.015<br>(0.031)[0.813]<br>h=18.0/18.0,<br>N=2143/928	−0.017<br>(0.034)[0.908]<br>h=18.0/18.0<br>N=1874/815
Systolic BP	−0.333<br>(0.589)[0.257]<br>h=10.0/10.0,<br>N=1194/768		−0.569<br>(2.065)[0.935]<br>h=10.0/10.0<br>N=679/421	−0.415<br>(0.477)[0.680]<br>h=14.0/14.0,<br>N=1894/980		−0.347<br>(1.736)[0.877]<br>h=14.0/14.0<br>N=1041/537	−0.227<br>(0.421)[0.332]<br>h=18.0/18.0,<br>N=2530/1131		0.361<br>(1.543)[0.604]<br>h=18.0/18.0<br>N=1412/626
Diastolic BP	−0.781<br>(0.795)[0.571]<br>h=10.0/10.0,<br>N=1194/768		−0.260<br>(1.283)[0.795]<br>h=10.0/10.0<br>N=679/421	−1.161<br>(0.667)[0.604]<br>h=14.0/14.0,<br>N=1894/980		−0.494<br>(1.070)[0.958]<br>h=14.0/14.0<br>N=1041/537	−1.007<br>(0.597)[0.194]<br>h=18.0/18.0,<br>N=2530/1131		−0.261<br>(0.952)[0.680]<br>h=18.0/18.0<br>N=1412/626
Body Mass Index	0.078<br>(0.503)[0.462]<br>h=10.0/10.0,<br>N=1138/737		0.528<br>(0.616)[0.872]<br>h=10.0/10.0<br>N=762/477	0.118<br>(0.415)[0.970]<br>h=14.0/14.0,<br>N=1810/935		0.625<br>(0.516)[0.563]<br>h=14.0/14.0<br>N=1166/605	0.124<br>(0.369)[0.926]<br>h=18.0/18.0,<br>N=2432/1078		0.520<br>(0.457)[0.338]<br>h=18.0/18.0<br>N=1571/702

Notes: This table present the impact estimates under several three specifications of a regression discontinuity design over the systolic blood pressure of respondents centred around 140 mmHg for ELSA. For males aged 50 and older in the HSE, the standardisation is done around 160 mmHg due to a different measurement protocol. It includes only those respondents aged 58 or younger at the time of the measurement who were not diagnosed with HBP or diabetes, and not taking BP-lowering medication. h presents the optimal bandwidth to the left/right of the cutoff, and N the corresponding number of observations effectively included.
Standard errors, in parenthesis, are derived from heteroskedasticity-robust nearest neighbour variance estimator with at least 3 neighbours. In the first set of brackets the conventional p-value is presented, while the second corresponds to the robust inference version derived by Calonico, Cattaneo, Titiunik (2014).

Table 3. The effects of information on potential hypertension status: polynomial order

Dependent Variable	p=1<br>Before BP test<br>$t=0$	 2 years<br>$t=1$	 4 years<br>$t=2$	p=2<br>Before BP test<br>$t=0$	 2 years<br>$t=1$	 4 years<br>$t=2$	p=3<br>Before BP test<br>$t=0$	 2 years<br>$t=1$	 4 years<br>$t=2$
**Panel A. Attrition**									
Missing this wave		-0.005<br>$(0.029)[0.805]$<br>h=14.8/12.7<br>N=2041/938	0.018<br>$(0.037)[0.540]$<br>h=13.0/13.5,<br>N=1737/979		-0.007<br>$(0.033)[0.778]$<br>h=23.3/21.5<br>N=3543/1232	0.026<br>$(0.044)[0.436]$<br>h=20.6/20.2,<br>N=3107/1213		0.055<br>$(0.047)[0.153]$<br>h=18.3/27.7<br>N=2732/1309	0.072<br>$(0.056)[0.132]$<br>h=22.1/25.5,<br>N=3409/1283
Already death by this wave		-0.003<br>$(0.005)[0.485]$<br>h= 8.2/ 4.8<br>N=977/395	0.000<br>$(0.008)[0.816]$<br>h=14.2/ 8.9,<br>N=1866/644		-0.002<br>$(0.005)[0.721]$<br>h= 9.4/ 8.1<br>N=1097/644	-0.009<br>$(0.013)[0.413]$<br>h=16.1/13.7,<br>N=2156/903		-0.008<br>$(0.006)[0.170]$<br>h=17.0/12.7<br>N=2156/863	-0.026<br>$(0.019)[0.148]$<br>h=17.2/19.0,<br>N=2323/1075
**Panel B. High Blood Pressure**									
Diagnosed HBP ever		0.057<br>$(0.027)[0.060]$<br>h=15.8/14.0<br>N=1874/846	0.040<br>$(0.035)[0.384]$<br>h=18.8/15.2,<br>N=2031/787		0.063<br>$(0.035)[0.075]$<br>h=22.4/16.3<br>N=2901/902	0.035<br>$(0.047)[0.563]$<br>h=20.5/22.3,<br>N=2315/922		0.060<br>$(0.042)[0.140]$<br>h=23.6/24.2<br>N=3011/1046	0.027<br>$(0.059)[0.622]$<br>h=23.6/25.2,<br>N=2644/950
Takes BP medication		0.060<br>$(0.020)[0.007]$<br>h=13.0/12.0<br>N=1477/769	0.034<br>$(0.025)[0.261]$<br>h=20.8/12.2,<br>N=2315/678		0.066<br>$(0.025)[0.023]$<br>h=18.3/15.9<br>N=2315/877	0.026<br>$(0.032)[0.613]$<br>h=24.3/14.2,<br>N=2743/744		0.066<br>$(0.028)[0.020]$<br>h=24.6/18.8<br>N=3116/961	0.025<br>$(0.037)[0.559]$<br>h=28.0/18.6,<br>N=3049/843
**Panel C. Health**									
Diagnosed Diabetes ever		0.010<br>$(0.011)[0.375]$<br>h=15.5/14.4<br>N=1874/846	0.017<br>$(0.019)[0.343]$<br>h=17.7/14.1,<br>N=1873/739		0.014<br>$(0.013)[0.263]$<br>h=18.6/26.2<br>N=2315/1065	0.021<br>$(0.024)[0.357]$<br>h=25.1/21.0,<br>N=2829/878		0.016<br>$(0.018)[0.341]$<br>h=19.9/25.9<br>N=2478/1054	0.026<br>$(0.029)[0.410]$<br>h=29.6/25.3,<br>N=3202/922
Diagnosed Heart Condition	-0.002<br>$(0.009)[0.787]$<br>h=15.0/13.4,<br>N=2039/977	0.018<br>$(0.017)[0.404]$<br>h=13.2/16.4<br>N=1603/903	0.036<br>$(0.021)[0.161]$<br>h=16.9/12.9,<br>N=1750/681	-0.001<br>$(0.012)[0.878]$<br>h=19.8/22.7,<br>N=2918/1244	0.014<br>$(0.022)[0.690]$<br>h=18.0/21.4<br>N=2156/1014	0.033<br>$(0.027)[0.265]$<br>h=17.3/24.6,<br>N=1882/926	-0.001<br>$(0.015)[0.820]$<br>h=24.6/23.3,<br>N=3660/1262	0.010<br>$(0.026)[0.761]$<br>h=21.5/27.4<br>N=2774/1074	0.038<br>$(0.033)[0.325]$<br>h=21.6/27.1,<br>N=2426/946
Self-reported GOOD health	0.016<br>$(0.034)[0.624]$<br>h=11.3/16.3,<br>N=1555/1094	-0.007<br>$(0.035)[0.941]$<br>h=12.1/18.4<br>N=1465/958	0.022<br>$(0.045)[0.630]$<br>h= 9.5/15.1,<br>N=882/766	0.013<br>$(0.043)[0.712]$<br>h=18.0/16.9,<br>N=2732/1094	-0.012<br>$(0.047)[0.747]$<br>h=16.4/18.5<br>N=1989/958	0.007<br>$(0.062)[0.928]$<br>h=13.4/13.8,<br>N=1390/704	0.026<br>$(0.046)[0.644]$<br>h=26.7/31.4,<br>N=3949/1345	-0.014<br>$(0.052)[0.934]$<br>h=24.7/22.1<br>N=3096/1022	0.009<br>$(0.067)[0.885]$<br>h=20.3/19.7,<br>N=2298/861
Systolic BP	-0.335<br>$(0.513)[0.518]$<br>h=12.0/13.2,<br>N=1739/980		0.026<br>$(1.624)[0.815]$<br>h=15.8/16.3,<br>N=1232/606	-0.460<br>$(0.690)[0.664]$<br>h=15.7/17.4,<br>N=2202/1131		-0.971<br>$(2.186)[0.584]$<br>h=17.4/22.5,<br>N=1412/687	-0.491<br>$(0.767)[0.584]$<br>h=23.4/22.3,<br>N=3543/1248		-1.499<br>$(2.420)[0.433]$<br>h=27.4/23.7,<br>N=2269/695
Diastolic BP	-1.193<br>$(0.652)[0.115]$<br>h=13.8/15.8,<br>N=1894/1065		-0.336<br>$(1.060)[0.765]$<br>h=12.9/16.3,<br>N=966/606	-1.103<br>$(0.869)[0.341]$<br>h=17.9/17.7,<br>N=2530/1131		-0.352<br>$(1.291)[0.836]$<br>h=20.2/22.7,<br>N=1715/687	-0.654<br>$(1.019)[0.689]$<br>h=23.2/19.7,<br>N=3543/1193		-0.382<br>$(1.498)[0.733]$<br>h=29.0/24.7,<br>N=2411/699
Body Mass Index	0.120<br>$(0.412)[0.618]$<br>h=13.9/14.7,<br>N=1810/980		0.771<br>$(0.548)[0.154]$<br>h=13.9/10.3,<br>N=1166/513	0.270<br>$(0.487)[0.565]$<br>h=22.2/21.9,<br>N=3282/1173		0.608<br>$(0.677)[0.470]$<br>h=17.3/19.4,<br>N=1571/741	-0.211<br>$(0.667)[0.589]$<br>h=21.6/23.2,<br>N=3146/1206		0.287<br>$(0.870)[0.965]$<br>h=19.3/23.4,<br>N=1793/780

Notes: This table present the impact estimates under several three specifications of a regression discontinuity design over the systolic blood pressure of respondents centred around 140 mmHg for ELSA. For males aged 50 and older in the HSE, the standardisation is done around 160 mmHg due to a different measurement protocol. It includes only those respondents aged 58 or younger at the time of the measurement who were not diagnosed with HBP or diabetes, and not taking BP-lowering medication. h presents the optimal bandwidth to the left/right of the cutoff, and N the corresponding number of observations effectively included.

Standard errors, in parenthesis, are derived from heteroskedasticity-robust nearest neighbour variance estimator with at least 3 neighbours. In the first set of brackets the conventional p-value is presented, while the second corresponds to the robust inference version derived by Calonico, Cattaneo, Titiunik (2014).

C Density test

The central assumption with the RDD design is that it is random whether a respondent is just above or below the cutoff. A describes how for individuals who are aware of their BP, this may not be apply. However, for individuals who are unaware of their BP problems, there is no reason for 'manipulation' of the running variable. One possibility is that nurses classified individuals with a BP below 140 mmHg as above that figure if they thought that an individual might benefit from the advice. It is possible to assess this is possibility with a standard density test. Here the Cattaneo, Jansson and Ma's test is implemented [1]. A non-parametric density estimator is obtained using local polynomials that avoids the pre-binning and is valid at boundary points. S3 Fig shows a graphic version of this test (implemented using *rddensity* routine) [2], where the difference in the densities is -0.3609 (bandwidths of 8.153 and 8.270 to the left and right), which cannot be rejected to be zero (p-value=0.7182).

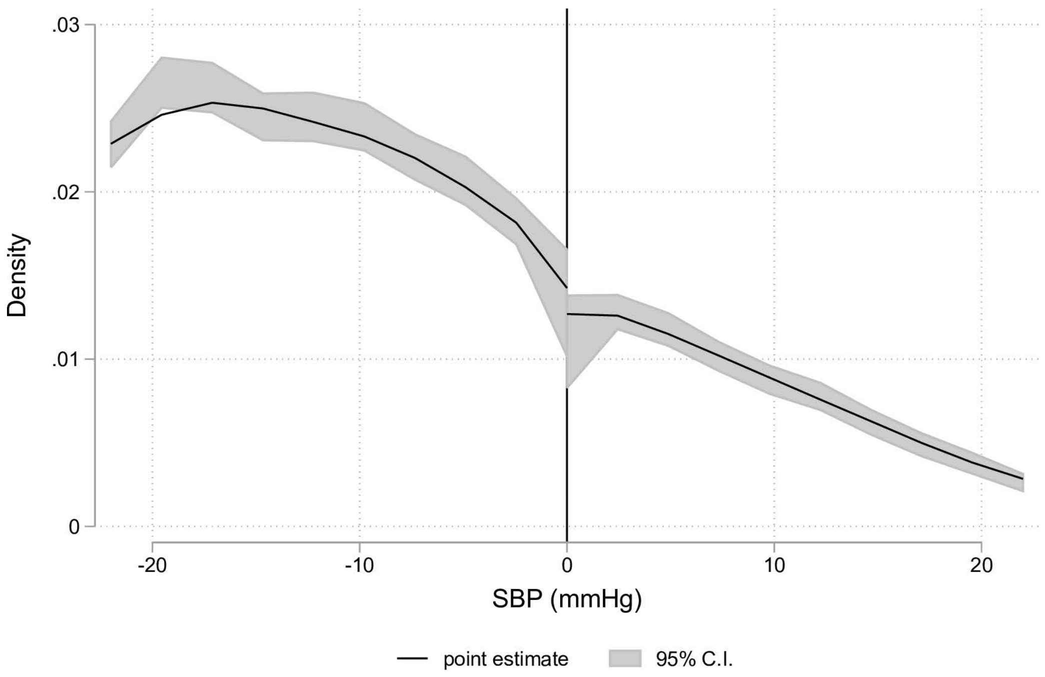

S3 Fig. Density discontinuity test

D Heterogeneous results

Based on the information model in the conceptual framework, one might expect the response to be stronger the worse the news about HBP are perceived. The same piece of information could be more negative because the underlying risk of CVD was greater owing to characteristics other than BP; it could also be because respondents considered their underlying health status to be much better. Table 4 presents heterogeneity at the 2 year window based on those two characteristics. Columns 1 and 2 show the 2-year results according to the 10-year CVD risk; columns 4 and 5 do so according to self-rated health. It is important to note that the cost of splitting the sample is obtaining less precise estimates about the jumps; it is likely that the
results were driven by pre-intervention imbalances.

The 10-year CVD risk index was calculated using the Framingham equation [3]. That is a standard risk calculator for individuals aged 30-74 years without prior CVD. It involves age, sex, smoking status, total and HDL cholesterol levels, SBP, and diabetes. The present study fixed SBP and diabetes status, so the variation comes from other variables. Respondents with a 10-year risk below 8% were defined as the low-risk group and those above that level as the high-risk group. This partition was done to maintain similar sample sizes in the two groups. As expected, even with a similar HBP diagnosis jump, the impact on medication was greater for the high-risk group. Moreover, for the high-risk group there was a jump in the prevalence of diagnosed diabetes.

HBP is bad news, but it is more likely that individuals with a high CVD risk expected such a result. For that reason, the split between individuals who reported that their health was *good* and *bad* might provide a better approach about the surprise factor with the advice. Those groups were balanced in the relevant outcomes prior to BP measurement. For respondents who considered their health bad, the point estimates for both jumps on diagnosis and medication use were at least twice the estimates for individuals who considered themselves in good health.

An additional exercise of particular interest is replicating the findings of [4] based on education and income. In essence, people with a higher income are more likely to adjust their lifestyle owing to their higher *value of life*, as indicated in the model in the conceptual framework section. However, with education, there is no similar prediction. Also, BP-specific outcomes were greater for respondents with an education up to college level; however, imprecise estimates made it difficult to make solid conclusions about lifestyle adjustment. In the case of income or wealth, the partition generated several pre-intervention differences, which also made it difficult to draw conclusions.

Table 4. The effects of information on potential hypertension status (1 wave ahead), by CVD-risk and Self-Rared Health

Dependent Variable	(1) CVD-risk Low	(2) CVD-risk High	(3) SR Health Bad	(4) SR Health Good
**Panel A. High Blood Pressure**				
Diagnosed HBP ever	0.051	0.083	0.139	0.036
	(0.047)[0.349]	(0.056)[0.158]	(0.073)[0.121]	(0.029)[0.254]
	h=16.9/11.6, N=909/257	h=16.4/12.2, N=509/263	h=19.3/11.4, N=409/124	h=17.0/13.1, N=1796/664
Takes BP medication	0.031	0.105	0.102	0.044
	(0.032)[0.377]	(0.040)[0.019]	(0.050)[0.099]	(0.021)[0.057]
	h=16.3/10.6, N=909/243	h=13.3/12.1, N=415/263	h= 6.8/11.0, N=107/124	h=18.2/11.2, N=1928/601
**Panel B. Health**				
Diagnosed Diabetes ever	0.002	0.056	−0.019	0.024
	(0.015)[0.958]	(0.024)[0.038]	(0.038)[0.657]	(0.010)[0.020]
	h=12.8/11.9, N=648/257	h= 7.3/15.5, N=218/300	h=19.3/12.2, N=409/132	h= 4.8/16.8, N=337/745
Diagnosed Heart Condition	0.040	−0.022	0.015	0.016
	(0.021)[0.104]	(0.047)[0.619]	(0.060)[0.843]	(0.015)[0.451]
	h= 4.8/14.0, N=164/300	h= 9.6/14.6, N=289/286	h=19.1/12.0, N=410/132	h=11.8/17.3, N=1104/772
Self-reported GOOD health	−0.064	0.066	−0.096	−0.003
	(0.056)[0.427]	(0.059)[0.300]	(0.101)[0.262]	(0.027)[0.994]
	h=12.1/13.1, N=643/287	h=13.6/16.0, N=411/309	h=17.9/ 9.2, N=353/107	h=16.4/12.0, N=1665/636

Notes: This table present the impact estimates under several three specifications of a regression discontinuity design over the systolic blood pressure of respondents centred around 140 mmHg for ELSA. For males aged 50 and older in the HSE, the standardisation is done around 160 mmHg due to a different measurement protocol. In includes only those aged 58 or younger at the time of the measurement. h presents the optimal bandwidth to the left/right of the cutoff, and N the corresponding number of observations effectively included.
Standard errors, in parenthesis, are derived from heteroskedasticity-robust nearest neighbour variance estimator with at least 3 neighbours. In the first set of brackets the conventional p-value is presented, while the second corresponds to the robust inference version derived by Calonico, Cattaneo, Titiunik (2014).

Table 5 presents the balance test for this segmentation of the sample. It shows that individuals just above the cutoff smoked on average fewer cigarettes in the high-CVD-risk group; those in the low-CVD-risk above the cutoff were less likely to consume alcohol twice a week or more. For that reason, it is important to be cautious with conclusions based on the heterogeneous impacts on lifestyle according to CVD-risk partition. However, for self-rated health, lifestyle and heart conditions are balanced below and above the cutoff within the partitions.

Similar partitions can be done in terms of education and wealth. This partitioning is interesting because it allows a comparison of results with Zhao et al [4], who found stronger values for high-income respondents and no differences for education. These are predictions from their model that they were able to test. For the education exercise, individuals were split according to their qualifications: those with high qualifications (equivalent to some college-level education) and others. In terms of wealth, the split was based on the median total benefit-unit wealth net of pensions (cutoff of 188.000 GBP in May 2005) at the $t + 1$ period (given that this precise measure was not available in wave 0).

Table 6 presents the balance test of the education and wealth partition, where individuals in the high wealth group were more likely to have been diagnosed with a heart condition. Accordingly, Table 7 presents the wave 1 results. In columns 1 and 2, coefficients for diagnosis of HBP and use of BP-lowering medication are greater for those in the low-education group. In columns 3 and 4, diagnosis and medication use jump coefficients and are similar in both partitions.

Table 5. The effects of information on potential hypertension status (Balance), by CVD-risk and Self-Rared Health

Dependent Variable	(1) CVD-risk Low	(2) CVD-risk High	(3) SR Health Bad	(4) SR Health Good
Diagnosed Heart Condition	-0.002	-0.004	-0.032	0.008
	$(0.002)[0.481]$	$(0.029)[0.978]$	$(0.037)[0.424]$	$(0.008)[0.445]$
	h= 4.5/ 5.1, N=193/207	h=15.0/12.1, N=538/312	h=17.8/11.1, N=434/152	h=10.5/17.6, N=1132/928
Self-reported GOOD health	0.008	0.061		
	$(0.046)[0.888]$	$(0.054)[0.290]$		
	h=13.9/15.2, N=858/391	h=14.1/16.9, N=538/369		

Notes: This table present the impact estimates under several three specifications of a regression discontinuity design over the systolic blood pressure of respondents centred around 140 mmHg for ELSA. For males aged 50 and older in the HSE, the standardisation is done around 160 mmHg due to a different measurement protocol. In includes only those aged 58 or younger at the time of the measurement. h presents the optimal bandwidth to the left/right of the cutoff, and N the corresponding number of observations effectively included.
Standard errors, in parenthesis, are derived from heteroskedasticity-robust nearest neighbour variance estimator with at least 3 neighbours. In the first set of brackets the conventional p-value is presented, while the second corresponds to the robust inference version derived by Calonico, Cattaneo, Titiunik (2014).

Table 6. The effects of information on potential hypertension status (Balance), by education and wealth

Dependent Variable	(1) Education Low	(2) Education High	(3) Wealth Low	(4) Wealth High
Diagnosed Heart Condition	0.001	-0.001	-0.020	0.022
	$(0.012)[1.000]$	$(0.001)[0.257]$	$(0.018)[0.244]$	$(0.011)[0.088]$
	h=15.1/15.8, N=1440/732	h= 5.7/ 7.6, N=207/204	h=15.4/11.4, N=896/340	h= 9.6/20.1, N=497/493
Self-reported GOOD health	0.007	0.017	-0.067	0.033
	$(0.044)[0.764]$	$(0.049)[0.967]$	$(0.059)[0.291]$	$(0.042)[0.502]$
	h=11.8/15.2, N=1038/735	h=13.5/11.1, N=635/270	h=13.9/12.8, N=773/366	h=12.1/14.8, N=722/418

Notes: This table present the impact estimates under several three specifications of a regression discontinuity design over the systolic blood pressure of respondents centred around 140 mmHg for ELSA. For males aged 50 and older in the HSE, the standardisation is done around 160 mmHg due to a different measurement protocol. In includes only those aged 58 or younger at the time of the measurement. h presents the optimal bandwidth to the left/right of the cutoff, and N the corresponding number of observations effectively included.
Standard errors, in parenthesis, are derived from heteroskedasticity-robust nearest neighbour variance estimator with at least 3 neighbours. In the first set of brackets the conventional p-value is presented, while the second corresponds to the robust inference version derived by Calonico, Cattaneo, Titiunik (2014).

Table 7. The effects of information on potential hypertension status (1 wave ahead), by education and wealth

Dependent Variable	(1) Education Low	(2) Education High	(3) Wealth Low	(4) Wealth High
**Panel A. High Blood Pressure**				
Diagnosed HBP ever	0.065	0.037	0.079	0.032
	(0.034)[0.102]	(0.044)[0.500]	(0.046)[0.076]	(0.037)[0.525]
	h=15.7/12.4, N=1197/522	h=19.8/12.7, N=894/246	h=18.5/10.1, N=1115/323	h=15.4/10.3, N=932/339
Takes BP medication	0.068	0.037	0.063	0.043
	(0.025)[0.024]	(0.035)[0.219]	(0.029)[0.050]	(0.024)[0.127]
	h=13.0/10.4, N=955/461	h=14.9/11.4, N=620/235	h=13.1/10.9, N=773/323	h=17.6/13.4, N=1074/396
**Panel B. Health**				
Diagnosed Diabetes ever	0.017	0.006	0.007	0.026
	(0.016)[0.281]	(0.009)[0.419]	(0.021)[0.590]	(0.013)[0.083]
	h=17.4/11.3, N=1372/490	h= 5.2/ 5.5, N=185/145	h=17.5/ 9.7, N=1030/305	h= 7.8/11.9, N=383/360
Diagnosed Heart Condition	0.033	−0.026	0.017	0.021
	(0.022)[0.208]	(0.018)[0.170]	(0.030)[0.704]	(0.016)[0.245]
	h=15.2/14.7, N=1198/576	h= 9.9/ 5.3, N=361/145	h=12.1/15.6, N=722/418	h=15.4/24.1, N=932/511
Self-reported GOOD health	0.016	−0.060	−0.041	−0.004
	(0.045)[0.676]	(0.050)[0.277]	(0.058)[0.544]	(0.040)[0.917]
	h=13.7/16.3, N=1018/613	h=12.3/13.7, N=516/254	h=13.3/13.9, N=763/383	h=12.6/15.1, N=720/427

Notes: This table present the impact estimates under several three specifications of a regression discontinuity design over the systolic blood pressure of respondents centred around 140 mmHg for ELSA. For males aged 50 and older in the HSE, the standardisation is done around 160 mmHg due to a different measurement protocol. In includes only those aged 58 or younger at the time of the measurement. h presents the optimal bandwidth to the left/right of the cutoff, and N the corresponding number of observations effectively included.
Standard errors, in parenthesis, are derived from heteroskedasticity-robust nearest neighbour variance estimator with at least 3 neighbours. In the first set of brackets the conventional p-value is presented, while the second corresponds to the robust inference version derived by Calonico, Cattaneo, Titiunik (2014).

D.1 Other cutoffs

A final exercise consisted of investigating discontinuities at other points in the sBP distribution. Given the index of SBP, it is possible to perform the same exercise but assuming the jump to be at values different other than 140 mmHg (160 mmHg for males aged 50 years and older at wave 0). This exercise has two advantages. First, it allows discarding random jumps on the dependent variables at many points of the SBP distribution (placebo exercise). Second, it allows testing whether there is an impact in providing more compelling advice at 160 mmHg (the clinical cutoff for hypertension stage 2). In this scenario, the phrasing of the feedback for the respondent is the same other than advising a visit to the family doctor within 2-3 weeks instead of 3 months. S4 Fig presents this exercise for the two main variables of interest according to the results: diagnosis of HBP and BP-lowering medication use. For males aged 50 years and older at wave 0, the mark was moved in 20 mmHg to reflect the original figures. The figure also provides the 95% confidence interval for each estimator. First, a jump on HBP diagnosis was not significant at this level for any specification (in the main results, the P value was 0.06). The graph shows one spike at 140 mmHg and another at 160 mmHg, but the latter was imprecisely estimated. Second, for medication, significant jumps are evident at 140 mmHg and at 160 mmHg. For 160 mmHg, the jump was close to 20 percentage points. For alcohol intake, on top of the base findings at 140 mmHg, there was also a large jump above the 160-mmHg level. The jump in the number of cigarettes per week was not significant at any point; however, it still presents two spikes: one at 140 mmHg and another at 160 mmHg.

Results close to 160 mmHg suggest stronger effects, but it is not possible to confirm that

inference. Fig 1 remind us that the sample size was conspicuously smaller at such levels of the running variable; that is the reason for the large standard errors associated with the jump estimators.

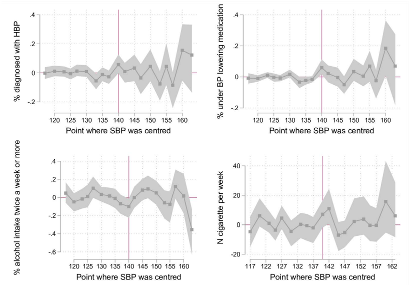

S4 Fig. Placebo jumps over standard SBP index

E Power calculations

Table 8 presents power calculations against impacts of 1 pp, 2.5 pp, and 5 pp for the binary variables; and ¼ standard deviation (SD), ½ SD, and 1 SD for the continuous variables. These numbers were calculated using [5] routine *rdpow*.

Detecting differential attrition is difficult (a test size of 0.316 for 5 pp, four years after the measurement), but the test seems adequate for mortality (0.68 for 2.5 pp, four years after the BP measurement). Concerning the main outcomes, it appears that with the given sample size, it is hard to detect impacts on HBP variables, showing the strength of the findings. As for health variables, the test has the power to detect changes of 5 to 10 mmHg on blood pressure and around 1.25 units of the BMI; while for self-reported good health and diagnosis of other conditions might be less suited. These limitations have been incorporated into the results and discussion section, which now is a separate sub-section of the document.

Table 8. Power calculations for the main specification

Dependent Variable	(1) Before BP test $t = 0$	(2) 2 years $t = 1$	(3) 4 years $t = 2$
**Panel A. Attrition. Power against** $1pp.\|2,5pp.\|5pp.$			
Missing this wave		$0.060\|0.114\|0.315$	$0.056\|0.087\|0.202$
		h=14.8/12.7, N=2041/938	h=13.0/13.5, N=1737/979
Already death by this wave		$0.473\|0.997\|1.000$	$0.169\|0.702\|0.999$
		h= 8.2/ 4.8, N=977/395	h=14.2/ 8.9, N=1866/644
**Panel B. High Blood Pressure. Power against** $1pp.\|2,5pp.\|5pp.$			
Diagnosed HBP ever		$0.061\|0.119\|0.332$	$0.056\|0.091\|0.219$
		h=15.8/14.0, N=1874/846	h=18.8/15.2, N=2031/787
Takes BP medication		$0.070\|0.180\|0.547$	$0.063\|0.136\|0.396$
		h=13.0/12.0, N=1477/769	h=20.8/12.2, N=2315/678
**Panel C. Health. Power against** $1pp.\|2,5pp.\|5pp.$			
Diagnosed Diabetes ever		$0.115\|0.461\|0.961$	$0.071\|0.187\|0.570$
		h=15.5/14.4, N=1874/846	h=17.7/14.1, N=1873/739
Diagnosed Heart Condition	$0.139\|0.580\|0.991$	$0.078\|0.230\|0.684$	$0.066\|0.155\|0.465$
	h=15.0/13.4, N=2039/977	h=13.2/16.4, N=1603/903	h=16.9/12.9, N=1750/681
Self-reported GOOD health	$0.057\|0.094\|0.234$	$0.057\|0.091\|0.221$	$0.054\|0.077\|0.159$
	h=11.3/16.3, N=1555/1094	h=12.1/18.4, N=1465/958	h= 9.5/15.1, N=882/766
**Panel D. Biomarkers. Power against** $1/4SD\|1/2SD\|1SD$			
Body Mass Index (SD=5.03)	$0.729\|0.999\|1.000$		$0.493\|0.973\|1.000$
	h=13.9/14.7, N=1810/980		h=13.9/10.3, N=1166/513
Systolic BP (SD=19.30)	$1.000\|1.000\|1.000$		$0.698\|0.999\|1.000$
	h=12.0/13.2, N=1739/980		h=15.8/16.3, N=1232/606
Diastolic BP (SD=11.53)	$0.957\|1.000\|1.000$		$0.616\|0.995\|1.000$
	h=13.8/15.8, N=1894/1065		h=12.9/16.3, N=966/606

Notes: This table present the power estimates under three alternative hypotheses of impact (1, 2.5, and 5 percentage points) for three specifications of a regression discontinuity design over the systolic blood pressure of respondents centred around 140 mmHg for ELSA. For males aged 50 and older in the HSE, the standardisation is done around 160 mmHg due to a different measurement protocol. It includes only those respondents aged 58 or younger at the time of the measurement who were not diagnosed with HBP or diabetes, and not taking BP-lowering medication. h presents the optimal bandwidth to the left/right of the cutoff, and N the corresponding number of observations effectively included.
Power calculations are robust bias-corrected.

References

1. Cattaneo MD, Jansson M, Ma X. Simple local polynomial density estimators. University of Michigan, Working Paper. 2017;.

2. Cattaneo MD, Jansson M, Ma X. rddensity: Manipulation testing based on density discontinuity. The Stata Journal (ii). 2016; p. 1–18.

3. D'Agostino RB, Vasan RS, Pencina MJ, Wolf PA, Cobain M, Massaro JM, et al. General cardiovascular risk profile for use in primary care the Framingham Heart Study. Circulation. 2008;117(6):743–753.

4. Zhao M, Konishi Y, Glewwe P. Does information on health status lead to a healthier lifestyle? Evidence from China on the effect of hypertension diagnosis on food consumption. Journal of health economics. 2013;32(2):367–385.

5. Cattaneo MD, Titiunik R, Vazquez-Bare G. Power calculations for regression-discontinuity designs. The Stata Journal. 2019;19(1):210–245.
